# Self-reported neurological symptoms in relation to CO emissions due to problem gas appliance installations in London: a cross-sectional survey

**DOI:** 10.1186/1476-069X-7-34

**Published:** 2008-07-01

**Authors:** Ben Croxford, Giovanni S Leonardi, Irene Kreis

**Affiliations:** 1 Bartlett School of Graduate Studies, University College London, 1-19 Torrington Place, London, WC1E 6BT, UK; 2 Chemical Hazards & Poisons Division, Centre for Radiation, Chemical and Environmental Hazards, Health Protection Agency, Chilton, Didcot, Oxon, OX11 0RQ, UK; 3 University of Wollongong, Wollongong, NSW 2533, Australia

## Abstract

**Background:**

Previous research by the authors found evidence that up to 10% of particular household categories may be exposed to elevated carbon monoxide (CO) concentrations from poor quality gas appliance installations. The literature suggests certain neurological symptoms are linked to exposure to low levels of CO. This paper addresses the hypothesis that certain self-reported neurological symptoms experienced by a householder are linked to an estimate of their CO exposure.

**Methods:**

Between 27 April and 27 June 2006, 597 homes with a mains supply of natural gas were surveyed, mainly in old, urban areas of London. Qualified gas engineers tested all gas appliances (cooker, boiler, gas fire, and water heater) and reported, according to the Gas Industry Unsafe Situations Procedure, appliances considered At Risk (AR), Immediately Dangerous (ID) or Not to Current Standards (NCS). Five exposure risk categories were defined based on measurement of CO emitted by the appliance, its features and its use, with "high or very high" exposure category where occupants were considered likely to be exposed to levels greater than 26 ppm for one hour. The prevalence of symptoms at each level of exposure was compared with that at lowest level of exposure.

**Results:**

Of the households, 6% were assessed as having a "high or very high" risk of exposure to CO. Of the individuals, 9% reported at least one neurological symptom. There was a statistically significant association between "high or very high" exposure risk to CO and self-reported symptoms compared to "no exposure" likelihood, for households not in receipt of benefit, controlling for "number of residents" and presence of pensioners, OR = 3.23 (95%CI: 1.28, 8.15). Risk ratios across all categories of exposure likelihood indicate a dose-response pattern. Those households in receipt of benefit showed no dose-response pattern.

**Conclusion:**

This study found an association between risk of CO exposure at low concentration, and prevalence of self-reported neurological symptoms in the community for those households not in receipt of benefit. As health status was self-reported, this association requires further investigation.

## Background

Prolonged exposure to concentrations of carbon monoxide that produce few symptoms and no clinical signs of acute poisoning, may produce effects on the central nervous system [1]. Occult CO poisoning has been described as cases of CO poisoning that may never come to the attention of a medical practitioner [2]. Case series of cardiovascular and neurological emergency admissions have been reported that screened for CO exposure and found evidence of this in 3 to 10% of the cases [3-6]. In patients that reach specialist clinical services after suspected exposure to low CO concentrations, many researchers record symptoms such as headache, impaired memory and concentration, and have documented them with neuropsychological testing [7,8].

Hampson [9,10] considers that in the United States, while fatal carbon monoxide poisonings have declined, non-fatal ones have remained high over the two decades from 1985–2002. A report by Wilson et al [11] found 4% of 300 homes in a 48 hour period exceeded 9 ppm CO, as an eight hour average. Recent research, by the author, first on 56 vulnerable homes around the UK, then on 270 vulnerable homes in the East London area found nearly 20% of homes exceeded the mean eight hour, World Health Organisation (WHO) ambient guideline for CO of 8.6 ppm. Further investigation by an engineer found many of these exceedances to be due to "problem" gas appliance installations. In both of these two research projects, gas fires and gas cookers were the most frequent source of the elevated CO emissions [12,13]. Few studies are available on the health effects of indoor exposure to CO.

This paper presents results from a study supported by the UK Health and Safety Executive (HSE) aimed at ascertaining the prevalence of unsafe gas appliances in residences in England [14], and examines the association between presence of a faulty gas appliance in a dwelling and the prevalence of self-reported neurological symptoms by the householder.

## Methods

A survey of gas appliances, conducted by qualified engineers was completed in August 2006, and a separate report describes the methods used for the selection of the homes and the individuals to be interviewed [14]. Homes from a total of about 80 postcode sectors in London were surveyed. The areas covered by the survey included mainly old urban areas and there were a high percentage (72%) of terrace homes in the sample; most of the homes were built between the world wars and very few newer homes were surveyed. The sample has a much higher percentage of homes (50%) with at least one person over 65 than the national average (20%). The sample of householders has a higher proportion of people on benefit (39.7%) than either London (28%) or national figures (24%).

A questionnaire was used to collect information on housing characteristics, gas appliances, behaviour in the building, and health. The health questions were developed in consultation with the team from the Medical Toxicology Unit at Guys Hospital, who have extensive experience in diagnosing patients with effects of carbon monoxide exposure. The questions were asked of one person per household, by the visiting gas engineer who was trained to ask the questions while carrying out the tests; this was considered likely to lead to the householder feeling relaxed and giving more accurate answers. The questions were asked at the same time as the gas appliances were being checked so any potential bias is expected to be minimised. Notably, the CO risk for the home was estimated after all appliance checks had been completed. The main health question was, "Have you experienced any of these symptoms WITHIN the home in the last week? Tick if yes", the symptoms were as given in table 1 and the prevalence of householder responses is also shown. Information on benefit status was collected to provide information on socio-economic status.

**Table 1:** Prevalence of self-reported symptoms as suffered in the last week within the house by the householder

Symptom	Sample % (n) (n, total = 597)
Headaches	7% (39)
Feeling faint	2% (9)
Feeling sick	3% (20)
Memory loss	2% (11)
Lack of concentration	2% (13)
Experienced confusion	1% (6)
No symptom information	1% (4)
1 or more symptoms reported	9% (53)
2 or more symptoms reported	4% (23)
3 or more symptoms reported	2% (11)

### Definition of Risk and Exposure Model

A CORGI (Council for Registered Gas Installers) qualified engineer inspected and tested all gas appliances (cooker, boiler, gas fire, and water heater), in each household. Under the Gas Industry Unsafe Situations procedure [15], gas engineers are required to make a notification where an appliance is considered At Risk (AR), Immediately Dangerous (ID) or Not to Current Standards (NCS). Appliances rated as AR or ID may not be associated with high CO emissions but with other installation issues also.

CO emissions were measured for each element of each gas appliance, but due to resource constraints of the project these methods did not follow the relevant British Standard completely [16]. The chief difference was for the measurement of CO emissions from gas fires, where a significantly shorter procedure than the British Standard was carried out [14]. However, all measurements taken provide a method for comparing emissions from different types of appliance and for assessing the risk of exposure to concentrations of CO greater than the WHO guidelines.

Five exposure categories were defined; these were based on risk categories that included a qualitative assessment of overall risk of the household to being exposed to concentrations of CO greater than the WHO guidelines [17]. This risk estimate was based on a measurement of the CO emitted by the gas appliance and several features of the appliance and its use. A summary of the criteria used to assess this risk and the prevalence of each risk category within the dataset, is given in table 2.

**Table 2:** Summary of risk criteria related to CO measurements, risk categories, and 5 exposure categories

Risk category	Exposure category	Risk criteria (all emissions of CO)	% (N)
Little or none	1	CO emissions from all appliances < 26 ppm	69% (409)
Low	2	Cooker emissions > 26 ppm Gas fire spilling any CO CO in boiler flue gases (room sealed) > 1000 ppm Boiler spilling any CO Poorly positioned flue terminal and flue gases of boiler > 52 ppm Boiler rated ID (any reason) and flue gases > 108 ppm	19% (111)
Medium	3	Cooker emissions > 52 ppm CO Gas fire spilling > 26 ppm CO Boiler spilling > 26 ppm Poorly positioned flue terminal and flue gases of boiler > 104 ppm	7% (42)
High	4	Cooker emissions > 104 ppm Gas fire spilling > 52 ppm CO Boiler spilling > 52 ppm Poorly positioned flue terminal and flue gases of boiler > 208 ppm	4% (22)
Very High	5	Cooker emissions > 208 ppm Gas fire spilling > 104 ppm CO Boiler spilling > 104 ppm Boiler flue gases (open flued) > 1000 ppm Poorly positioned flue terminal and flue gases of boiler > 416 ppm	2% (13)

Total			100% (597)

### Statistical analyses

Logistic regression analyses were conducted using the statistical software package Stata v8.2. For some of the analyses, it was necessary due to numbers in each category, to combine exposure levels 4 and 5 to form a "high exposure level", giving 4 risk categories. For each category, odds ratios for the prevalence of self-reported symptoms were computed (using level 1, "little or no risk" as the baseline category in all cases). Models were developed to estimate the unadjusted odds ratios, and also those controlling for benefit status, presence of pensioners, and number of residents. The information on these three variables was considered relevant to the estimate of socio-economic status. An additional model was also developed considering benefit status as a clustering variable. Finally, the role of exposure-confounder interaction was evaluated by fitting models estimating health effects separately for levels of benefit status, presence of at least a pensioner, and number of residents in the household.

## Results

The prevalence of selected self-reported symptoms suffered by the householder in the last week within the house is shown in table 1.

Table 2 shows the prevalence of households for each CO exposure risk category, 6% of homes are estimated as having a high or very high risk of being exposed to CO concentrations greater than the one hour WHO guideline for CO in ambient air, while 69% are assessed as having little or no risk of exposure to CO.

The numbers of homes with gas appliances having some sort of problem and that were rated as at risk (AR) or immediately dangerous (ID) are given in table 3. Twenty two per cent (131/597) of homes were found to have at least one appliance rated as AR or ID. The prevalence of householders having at least one symptom is much higher (15%) for those households with at least one AR or ID appliance, than if no problem appliance is present (7%). When the risk ratio was computed for the risk of a householder having at least one self-reported neurological symptoms for householders with at least one gas appliance rated AR or ID, compared to those residents in households with no appliance thus rated, it was found to be statistically significant, risk ratio = 2.40, (95%CI: 1.41, 4.08).

**Table 3:** Health symptoms and appliances rated as AR or ID

	No symptoms reported %(n)	Any symptom reported %(n)	No symptom information	Total of each row
No appliances rated as AR or ID	92% (431)	7% (33)	0% (2)	100% (466)
At least one appliance rated as AR or ID	83% (109)	15% (20)	2% (2)	100% (131)
Total of column	90% (540)	9% (53)	1% (4)	100% (597)

Table 4 shows that households reporting more symptoms have a higher prevalence of having at least one appliance rated as AR or ID.

**Table 4:** Health symptoms and problem appliances

Number of symptoms reported	Total households reporting n symptoms	% of people reporting n symptoms with at least 1 appliance rated as AR or ID
0	543	20% (109)
1	27	33% (10)
2	12	42% (5)
3+*	11*	45% (5)*
No symptom information	4	50% (2)
Totals	597	22% (131)

*Addition of 3 or more symptoms to give 3+ symptoms

Considering the estimated risk of CO exposure and self-reported symptoms, the fourth column of table 5 shows a higher prevalence of self-reported symptoms for those households with higher assessed risk of CO exposure.

**Table 5:** Prevalence of CO risk categories across the sample and health symptom response by risk category

CO Risk category	Prevalence in sample % (n)	No health symptoms reported	1 or more health symptoms reported	No symptom information
A_Very High	2% (13)	77% (10)	23% (3)	0% (0)
B_High	4% (22)	82% (18)	18% (4)	0% (0)
C_Medium	7% (42)	81% (34)	17% (7)	2% (1)
D_Low	19% (111)	89% (99)	10% (11)	1% (1)
Little or no risk	69% (409)	93% (379)	7% (28)	0% (2)
Total	100% (597)	90% (540)	9% (53)	1% (4)

Combining the two highest risk categories into one high risk group and calculating the odds ratios for symptom risk for each exposure risk category, gives a dose-response pattern (see table 6), this pattern is apparent in the unadjusted odds ratios and remains after adjusting for confounders. When the data is further split into two groups; those either in receipt of benefits or not, there is a clear distinction, with the group "not in receipt of benefit" showing a strong dose-response pattern and the "in receipt of benefit" showing no such pattern.

**Table 6:** Risk of reported health symptoms for 4 levels of carbon monoxide exposure likelihood

CO exposure	Unadjusted odds ratios (95%CI: Low, High)	Odds ratios version 1* (95%CI: Low, High)	Odds ratios version 2* (95%CI: Low, High)	Odds ratios version 3* (95%CI: Low, High)	Odds ratios version 4* (95%CI: Low, High)
Little or no risk	1.00	1.00	1.00	1.00	1.00
D_Low	1.50 (0.72, 3.13)	1.46 (0.70, 3.05)	1.46 (0.70, 3.04)	0.54 (0.15, 1.95)	3.08 (1.20, 7.91)
C_Medium	2.79 (1.13, 6.85)	2.82 (1.14, 7.02)	2.81 (1.14, 6.96)	2.22 (0.66, 7.50)	3.22 (0.84, 12.4)
B_High or A_Very High	3.38 (1.36, 8.43)	3.25 (1.29, 8.22)	3.23 (1.28, 8.15)	0.98 (0.21, 4.63)	9.28 (2.78, 30.98)

*Version 1: adjusted for benefit, number of residents, and presence of pensioners

*Version 2: using "benefit recipient" as clustering variable, and adjusted for "number of residents" and presence of pensioners

*Version 3: in the subgroup who are "in receipt of benefit"

*Version 4: in the subgroup who are "not in receipt of benefit"

## Discussion

The presence of a gas appliance rated as AR or ID, or if the assessed risk of exposure to CO is high this does not necessarily mean that the householder is actually exposed to high levels of CO. The concentration of CO that the householder is exposed to also depends on their use of their appliances, the available ventilation and also their behaviour around the appliance itself. So, the risk exposure assessment is an indication of possible risk rather than actual risk.

In general, the self-reporting of symptoms by study participants can be open to the possibility of over-reporting by individuals concerned about the effects of CO, leading to biased estimate of CO effect. This appears unlikely in this study as the visiting engineer was primarily checking the appliances and the health question was asked as one short question in a 1 page questionnaire. Neither engineer nor householder knew the estimated CO risk category as this was only established after the health data collection was completed.

In the questionnaire responses we gathered information on whether the household is in receipt of any benefit. This is self-reported information and may not be accurate; however we have no other information on socio-economic status. We believe that benefit status is strongly related to socio-economic conditions, for example smoking behaviour can be predicted by neighbourhood deprivation measures [18]. We consider that by controlling for benefit status at household level this is likely to at least partially account for smoking status as well as socio-economic status in the analysis.

Table 6 shows that the estimated effect of CO exposure on symptoms is only marginally reduced by adjusting for; benefit status, "presence of at least one pensioner", and "number of residents in the household". A separate, fixed effects model, considering "benefit status" as a clustering variable, also produced a very small reduction in effect that remains significant for Medium and for High risk of exposure to CO (table 6). We believe the detection of a clear, dose-response pattern increases the likelihood that the association seen may be causal.

The role of "benefit status" as a risk factor for self-reported neurological symptoms was also considered, both unadjusted, and adjusted for; CO exposure, "presence of pensioners", and "number of residents". We found "benefit status" to be a risk factor for self-reported neurological symptoms with an adjusted OR of 2.46 (95%CI: 1.13, 5.35). Of the interactions studied, this was the only significant one found (p = 0.0429). Separate estimates were therefore produced for the subgroup who reported being "in receipt of benefit" and those "not in receipt of benefit" (table 6). While the trend, of increasing CO risk leading to increased likelihood of reporting neurological symptoms, seems clear in all of the previous analyses considering the whole dataset, this pattern is only clearly present in the subgroup "not in receipt of benefit". In view of the demonstrated effect of benefit status on symptom risk, and the likelihood that as a group those "in receipt of benefit" are more likely to be poorer and also to be smokers, this may account for the lack of a clear effect of CO exposure on self-reported symptom risk in this subgroup.

It is important to note that this survey was carried out in summer; it is likely there would be increased ventilation and decreased use of gas appliances at this time compared with a survey carried out during the winter months, wintertime exposure could well be greater.

A simple extrapolation can be made from this dataset to estimate the potential numbers affected by CO related neurological symptoms in England. From table 2, 22% of all homes were found to have an AR or ID appliance; this corresponds to as many as ~4.6 million homes in England Scotland and Wales [14]. The difference in prevalence between the group of homes with an AR or ID appliance (15%) and the baseline (7%) leads us to estimate that a total of 8% of all self-reported neurological symptoms in homes with an AD or IR appliance could be due to CO exposure suggesting that as many as ~2% (22% * 8%) of all homes on gas could be affected. However, 25% of homes in the UK are in receipt of benefit [19], and a similar percentage of the population are smokers, in these homes symptoms may be due to issues related with smoking or with low socio-economic status. Considering these points we then suggest that ~1% of all homes with gas appliances might have householders suffering symptoms such as those reported here, caused by exposure to carbon monoxide from their gas appliances. Further research is needed to investigate this association.

## Conclusion

This study is the first to quantify the association between risk of CO exposure at low concentration, and the prevalence of self-reported neurological symptoms in the community. The presence of an unsafe gas appliance installation appears to be linked to an increased risk of suffering at least one of the six neurological symptoms listed in table 1, OR = 2.40 (95%CI: 1.41, 4.08).

Detailed analysis, using the estimated risk of a householder being exposed to concentrations of CO greater than the one hour WHO guideline for ambient air, shows an even closer link to reported symptoms, (for Low risk OR = 1.50, Medium risk OR = 2.79, High risk OR = 3.38). Using information likely to predict socio-economic status, reduces these overall risk ratios very slightly but more importantly when the dataset is split into two groups; those either "in receipt of benefit" or "not in receipt of benefit", this trend disappears in the former group. In the group "not in receipt of benefit" the trend is much stronger than for the dataset as a whole, (for Low risk OR = 3.08, Medium risk OR = 3.22 but not statistically significant, High risk OR = 9.28).

For those "not in receipt of benefit" the effect of CO exposure seems clear, indicating the possibility that for all groups, CO exposure in the home is a risk factor for neurological symptoms, simple extrapolation of the results here suggest the problem may be a significant one for the general population. The possibility that exposure to low concentrations of CO may give rise to health effects has been suggested before [1,2], and the present study provides direct support for this.

Possible bias associated with self-selection of patients attending emergency services or specialist clinics was avoided in this study. The effect of confounding variables such as diet or smoking was not included.

Direct measurement of personal exposure to CO will be addressed in future research, but we consider that the association reported here, is likely to be causal. Given the possible number of individuals with such symptoms attributed to CO exposure, there are large public health implications if they could be confirmed.

Considering the morbidity burden of symptoms as common as headache and loss of memory, the possibility that even a small fraction of them could easily be prevented by interventions on the use and operation of gas appliances deserves further consideration.

## Competing interests

The authors declare that they have no competing interests.

## Authors' contributions

BC and GL conceived the project, BC set up and managed all aspects of the project, he also managed the quality control and analysis of the data, GL and IK carried out some of the statistical analysis and helped draft the manuscript. All authors read and approved the final manuscript.
